# Business of radiology/radiologist in business

**DOI:** 10.4103/0971-3026.76069

**Published:** 2011

**Authors:** Smiti Sripathi

**Affiliations:** Department of Radio-diagnosis and Imaging, Kasturba Medical College, Manipal University, Karnataka, India E-mail: smitis11@hotmail.com

Dear Sir,

Your editorial “Business of radiology” in Nov 2010 issue is truly thought provoking.[1] As a faculty in radiology in a medical college, I ponder at times as to why privileged students who can opt for any other branch of medicine choose radiology. The myth about radiologists minting money is a universal phenomenon. By the time the student and his/her parents realize that this may not be true, it is often too late. The money invested in a radiology seat is not easy to recover. It goes without saying that without exceptional circumstances it is not easy to earn that kind of money quickly and it takes time for a young radiologist to gain experience and to work independently. It is easier said than done.

As a salaried radiologist, I am often rebuked by colleagues from other disciplines for staying in the safe confines of a college and for not going into private practice, thereby losing out on the potential to earn a good sum of money. What they fail to understand is that apart from the infrastructure, human resources, accounting, etc., disciplines about which many of us have absolutely no idea, it also needs a lot of courage to venture into an unknown territory. One big hitch that stops people from being on their own is the lack of knowledge of these non-medical issues that are taught in a business school but not in a medical college. Also, there is no definite correlation between being good in your profession and earning more money. While it is true that there are a few radiologists who do earn the kind of money that people think we earn, this is not a universal truth.

One must also not forget that a lot of students from colleges charging such hefty fees pass out promptly, and in a good majority of these colleges, the quality of the radiologist produced is questionable. So even though there should be some emphasis on learning the “business of radiology”, this should not be at the expense of the radiology training.

## References

[1] Jankharia B (2010). The business of radiology. Indian J Radiol Imaging.

